# On the pathway of changing emotions—a qualitative study of emotional changes in breast cancer patients from the first detection of the tumor to breast surgery

**DOI:** 10.1186/s12905-026-04776-6

**Published:** 2026-08-14

**Authors:** Tünde Lévai, György Lázár, Attila Paszt, Zsolt Simonka, Zoltán Horváth, Aurél Ottlakán, Melinda Látos

**Affiliations:** 1 Department of Surgery, Albert Szent-Györgyi Health Centre, University of Szeged, 8, Semmelweis Street, Szeged, 6725 Hungary; 2 Institute of Psychology, Department of Personality, Clinical and Health Psychology, University of Szeged, 2, Egyetem Street, Szeged, 6722 Hungary

**Keywords:** Breast cancer, Illness trajectory, Emotional changes, Emotional Graph of Illness Trajectory, Qualitative study

## Abstract

**Background:**

The trajectory of breast cancer is a dynamic experience, both physically and emotionally, and despite the same diagnosis, the emotional reactions to events during the healing process are quite individual. Therefore, to support the coping processes with the disease effectively in psychological practice with cancer patients, it is essential to explore the emotional reactions of patients and to gain insight into their emotion regulation processes. Our study aimed to explore different types of emotions that accompany the disease process and the impact of disease events on changes in the intensity of emotions.

**Methods:**

We applied an exploratory, descriptive, phenomenological approach, and through purposive sampling, we involved 42 female participants (mean age 53.44, SD: 8.80) who had undergone breast surgery to conduct face-to-face semi-structured interviews based on Emotional Graph of Illness Trajectory, as a graphical elicitation technique. Interview data were analysed using content analysis involving two independent coders.

**Results:**

73.8% of the women with breast cancer reported one dominant emotion associated with their disease process, while 59.52% reported only negative emotions connected to their disease pathway. The most frequent negative emotion was fear, and the most frequent positive emotion was hopefulness. Our most outstanding results—such as the association of positive meanings linked to the course of the illness with positive emotions—suggested that patients' positive mental representation of the illness and healing process could have contributed to the adaptive emotion regulation.

**Conclusions:**

Presumably, the development and change of the emotion associated with the disease process was determined by the patient's perception of the disease and the healing process, on the one hand, and the meaning attributed to the event, on the other hand. Our results highlight the importance of exploring the emotional aspects accompanying the disease process, as the emotional perspective provides insights into the emotional side of coping style during the disease process. The Emotional Graph of Illness Trajectory can help to restructure and re-evaluate the experience of illness and thus restore the disconnectedness of life history, which can lead to a psychological process that adaptively contributes to reducing the traumatic impact of disease.

**Trial registration:**

ISRCTN, ISRCTN10290883. Registered 25 April 2024 – Retrospectively registered, https://www.isrctn.com/ISRCTN10290883

**Supplementary Information:**

The online version contains supplementary material available at https://doi.org/10.1186/s12905-026-04776-6.

## Background

### The physical and psychological effects of malignant breast cancer

When faced with a diagnosis of breast cancer, nearly without exception, all women experience an intense emotional reaction and this situation triggers an adaptation process involving a complex variety of emotions [1–3]. The diagnosis of breast cancer is almost inseparable from depressive and anxious reactions, coupled with feelings of helplessness, hopelessness and shame, which can underpin a loss of motivation to cope effectively with the disease, however, many patients adopt a resilient attitude [3–5]. Based on the results of interviews on the physical and psychological effects of breast cancer in the qualitative exploratory study by Iddrisu and colleagues [1], a significant proportion of women cried, felt depressed, and were emotionally unstable when they were told of their diagnosis. According to their results, patients reported severe physical weakness due to the effects of the treatments, resulting in an inability to carry out daily activities and negative changes in body image, which may have led to depressive symptoms [1]. Lemieux, Maunsell, and Provencher [6], conducted a review of the effects of hair loss on female cancer survivors and found it to be one of the most disruptive side effects of chemotherapy [6]. Liu and colleagues [5] examined psychological and emotional changes in patients before and after breast surgery. They found that levels of depression and anxiety increased preoperatively, but were alleviated postoperatively [5].

Body image changes are common after breast cancer. They may include physical alterations from surgery, such as breast loss, scarring, and asymmetry, as well as side effects of treatment like hair loss, weight fluctuations, lymphoedema, and skin texture changes. These changes can cause feelings of loss, sadness, reduced self-esteem, and dissatisfaction with one's own body; moreover, they are strongly associated with a loss of femininity [7–10]. As a result, sensations related to body awareness that were previously ignored often become more prominent, which can increase fears of cancer recurrence [11]. Developing a positive body image depends on the individual's experiences with their illness and their ability to address body- and emotion-related issues, which helps form a new, positive body image after cancer [10].

Past research suggests that innovative and combined intervention methods, such as physical exercise or web-based interventions, are effective in promoting positive body image and preventing self-fragmentation [12]. Recent studies also emphasize the importance of mindfulness- and self-compassion-based psychological interventions, as they foster kindness and reduce self-judgment—key elements in enhancing the well-being of women with breast cancer [10, 13, 14]. In addition, beneficial effects have been found regarding the impact of Virtual Reality intervention on interoceptive abilities and improvement of emotional well-being in breast cancer survivors [15].

Furthermore, like most cancerous diseases, breast cancer is associated with a loss of control over the body [16]. When facing cancer, the continuity of life is disrupted, the narrative coherence of the life story is broken, potentially resulting in a loss of emotional balance and an uncontrollable impact on emotion regulation. The diagnosis makes it difficult to adapt and cope with the short and long term psychological distress and physical symptoms of the illness [17–19].

The trajectory of cancer is a dynamic experience, both physically and emotionally, which varies significantly between diagnosed individuals even with the same type of cancer [20–22]. Emotion regulation strategies can significantly influence patient attitude towards the disease, therapeutic engagement, resource mobilisation, and thus multiple dimensions of coping with the disease [22, 23]. Specifically, emotion regulation is “the processes by which individuals influence which emotions they have, when they have them, and how they experience and express them” ([24]: p. 275). In their theoretical framework of Affect Regulation in Cancer, Kangas and Gross [22, 25] distinguish five families of cyclically related and interacting emotion regulation strategies: situation selection—involves choosing situations based on their likely emotional impact; attention deployment—strategic and conscious control of mental focus toward certain stimuli or away from others; cognitive change—ability to change internal thoughts, attitudes, and emotions; response modulation—transformation and modification of responses to stimuli, and situation modification—strategy for altering an existing situation to change its emotional impact.

The recognition of emotional distress and potential mood disorders is crucial for health prevention [26]. For oncology care professionals, especially psychologists, it is vital to develop and utilise tools that help patients recognize and express their emotions, share their fears, and regain control of their lives [27–30]. Despite evidence of many emotional and mood problems, cancer patients rarely have the opportunity to directly and spontaneously express their concerns and emotions in a medical consultation context. Their emotional signals are usually embedded in discussions about medical information and are often less openly revealed [3].

### Objective

There is a large body of research exploring the psychological consequences of diagnosis, treatments, and their side effects among breast cancer patients, but there is a lack of literature on the impact of events on the emotional state of patients during the illness trajectory and recovery process, narrated by the patient as a structured perspective. Therefore, our exploratory descriptive study aimed to explore the event-related emotional experiences in the postoperative state, during the illness trajectory of women with breast cancer.

## Methods

### Study design

The study applied an exploratory descriptive, phenomenological approach with open-ended questions in semi-structured interviews in the form of face-to-face interviews to qualitative research to explore, understand, and describe the emotion-related experiences of women with breast cancer, from the first detection of a breast tumor until the breast surgery. Exploratory descriptive design is a research design that aims to describe phenomena by exploring them from the participants' perspective; a research method that first uses flexible, qualitative approaches to explore a less-understood topic, after which more structured methods are applied to describe the results in detail [31]. To conduct qualitative research, the Emotional Graph of Illness Trajectory was used as a visual elicitation technique that generates visual and verbal data [32]. The study followed the content analysis methodological framework during the data collection and data analysis and interpretation of findings. This study followed the Consolidated Criteria for Reporting Qualitative Research (COREQ) guidelines [33].

### Study setting

The study was conducted at a surgical clinic [location masked for blind review]. Purposive sampling was used to gain the most accurate and comprehensive understanding of the experiences we aimed to investigate. Breast cancer patients were recruited from breast surgery wards within five days of their operation. The personal recruitment was carried out by psychologists conducting the interviews. Following the personal consultation in the patient room, the patients were informed and then interviewed. Data collection and data analysis took place simultaneously. The study sample consisted of 42 female participants, resulting in 42 interviews, with saturation reached. Saturation is the point when no new or repetitive responses emerge in participant reports [34]. Data saturation was determined when no new codes or main themes emerged during the last few interviews. Each subject was assigned an identification number from 1 to 42 to ensure anonymity.

Age of participants ranged between 40–77 years. The inclusion criteria in the study sample was: age over 18 years, diagnosis of non-metastatic malignant breast cancer (stage I-III), surgery performed in the last five days for breast cancer, and literacy skills to read and write. Exclusion criteris was: any current, or historic, severe psychiatric or neurological illness experienced by the patient, which information was screened using the medical documentation system. Descriptive data presented in Table 1.

**Table 1:** Clinical and sociodemographic characteristics of participants

**Clinical data**	**Number of participants (% of the study sample)**
Diagnosis of malignant breast cancer	42 (100%)
Type of breast surgery	
Mastectomy	15 (35.72%)
Mastectomy with reconstruction	2 (4.76%)
Lumpectomy	25 (59.52%)
Neoadjuvant chemotherapy	12 (28.57%)
Time from diagnosis (mean, years)	0.63 years = 7.7 months (SD: 0.96; range: 0.08–2.0 years)

**Sociodemographic data**	**Number of participants (% of the study sample)**
Age (years)	53.44 (SD: 8.8)
Gender (Female)	42 (100%)
Employment activity	
Active worker	20 (47.61%)
On sick leave	12 (28.57%)
Disability pension	1 (2.38%)
Unemployed	2 (4.76%)
Retired	7 (16.66%)
Domicile	
Live in the capital	0 (0%)
Live in city	30 (71.43%)
Live in village	12 (28.57%)
Marital status	
Single	1 (2.38%)
Couple	6 (14.29%)
Married, living with spouse	28 (66.66%)
Married, not living with spouse	0 (0%)
Divorced	4 (9.52%)
Widow	3 (7.1%)
Level of education	
Primary qualifications	2 (4.76%)
Intermediate qualifications	23 (55.76%)
Higher education degree	17 (39.48%)

### Data collection procedure

Data were collected from breast cancer patients, recently having breast surgery. Patient contacts and data collection were conducted by authors. Research team members, who were clinical health psychologists and breast surgeons at the surgical clinic where data was collected, participated in training in qualitative research, two of whom conducted the interviews. Female interviewers had postgraduate degrees in clinical health psychology. The interviewer had no relationship with the participants prior to the study. The participants were only aware about the occupation and prior clinical experience with breast cancer patients of the interviewers.

The test enrollment period of our study ran for 12 months, from September 2020 to September 2021. Participation in the study was voluntary and conducted with written informed consent attained from patients. The research was carried out in accordance with the Declaration of Helsinki and with the approval of the relevant ethics committee (due to double anonymity, the name of the authorising committee and the approval number are not indicated) [35].

The single, face-to-face test sessions were carried out within five days post-surgery, adapting to the constraints of the hospital stay and considering the patient's physical condition. The interviews lasting 30–60 min, were conducted in a quiet room with comfortable environment to ensure that the interview process would not be disturbed. During interviews, the researcher was cautious not to project her experiences on the participants, writing down exactly what the participants portrayed. The Emotional Graph of Illness Trajectory served as the interview guide. Ten percent of interviews were repeated, in order to verify temporal reliability. During the interviews, the interviewers applied contemporaneous researcher notes on participant answers, carried out according to the guide of Emotional Graph of Illness trajectory. The marking sheet used during the study and the data attained are functionally equivalent to field notes, since this verbal data was included in our content analysis; however, for the same reason, we did not specifically use field notes. We prepared transcripts of the interviews, although these were not sent to study participants for review.

### The observed psychological factors

Before the interview, a block of sociodemographic questions was designed to assess respondent age, gender, employment status, domicile, education, and marital status*.* In addition, we recorded the medical data according to diagnosis, type of neoadjuvant therapy, type of breast surgery (mastectomy, mastectomy with reconstruction, lumpectomy), and elapsed time in years since diagnosis.

The *Emotional Graph of Illness Trajectory* visual elicitation technique was used to explore the predominant emotion in the disease process, the relationship between events in the disease process and this emotion, and the form and explanation of emotional state changes [32]. The technique may discover the dominant emotion characterising the disease process and the emotional impact of disease-related events [32, 36]*. *The technique used by our research team was based on the graphical procedure of Thygesen and colleagues [36]: a graphical elicitation technique with a timeline structure using a grid-graphic tool, including a horizontal axis representing events in chronological order and a vertical axis with a 0–100% coverage to assess intensity of experienced emotion.

During the interview, the patient was first asked to name the most potent emotion they had experienced during the illness. In the next step, the patient was asked to list, in chronological order along the horizontal axis, the emotionally significant events in the course of the disease and then to rate, on a scale of 0–100%, the strength of the emotion experienced during these events along the vertical axis. It was up to the patient to construct the graph during the test session. While the patient was creating the graph and sharing information about it, the investigator recorded this information on a marking sheet, developed by our research team. During the interview, the patient is primarily asked to try to name and graphically represent one dominant emotion. However, the graphical interviewing technique allows subjects to name and graphically represent more than one emotion.

Compared to the original procedure and test-taking sheet of the elicitation technique created by Thygesen and colleagues [36], we made some modifications and innovations to our enhanced tool [32]. (1) the horizontal axis of our instrument does not contain predefined standard event characteristics of the disease process, (2) the patient determines the onset of the disease process, and (3) the grid covers the entire area between the two axes in the form of equally sized squares. The grid area was provided to the patient in A/4 sheet size, covering the entire sheet, with instructions included. In addition, we created a marking sheet on which the investigator recorded information related to the emotion selected by the patient (reason for the emotion, name of the associated events, description of the content, and the intensity of the emotion experienced during the events (Additional file 1: Supplementary material). Changes to the instrument were motivated by our research team's objective of using it to understand the patient's subjective assessment of the onset of illness. In addition, changes were made to understand the self-constructed illness story, structured around self-reported events that the patient considered significant. Therefore, we did not include formal events on the horizontal axis of the graph. A grid area covering the entire area between the axes has been included to help plot the graph and mark the numerical values more accurately.

The Emotional Graph of Illness Trajectory was further supplemented with a post-test interview, based on the interview questions from the original method [36]. Post-test questions were designed to identify which other individual or socio-environmental factors, beyond the impact of the events related to the illness described, might have played a role in the change in the intensity of emotion. The post-test interview questions were "*What could have caused the rise/fall of the graph?"; "If there were turning points, what caused them?"; "What did the patient do to change the low/high points?*". The investigator also recorded the answers in the space provided on the marking sheet.

### Data analysis

During the interview session of the Emotional Graph of Illness Trajectory, the researcher recorded these following data on the marking sheet: the dominant emotion of the illness trajectory and its justification, the main events of the illness process named by the patient, the content of the event about the named emotion, the intensity of the emotion related to the given event expressed in percent, and finally the answers to the questions asked during the post-test. The theme of the interview was therefore the emotional changes accompanying the assessed course of the illness, which emerged during the course of the interview. The data generated by the patient was all visual information: on the template, the patient described in chronological order along the horizontal axis the events that are considered significant for him/her, ploted the emotional intensity associated with these events along the vertical axis, and then ploted the intersection of these values between the axes to produce a graph of the change in emotional state. A verbatim electronic transcript of the notes taken was made and this digital version was used for data analysis.

The data recorded by the researchers and the visual data created by the respondents are presented descriptively and subjected to thematic content analysis. Both the written data and the visual data were carefully reviewed several times by each researcher. After data interpretation, summarised and described results are exclusively interpreted and described by the authors. Only the named emotions are included in this category. However, in the analysis of data that require interpretation and description, two independent coders were included. The thematic content analysis aimed to understand the emotions associated with the disease process in breast cancer patients studied, and to understand how changes in experienced emotions—increased or decreased in intensity—could be associated with events during the disease process.

In the thematic content analysis, recorded information on the marker sheet was processed together with the visual content by independent coders. For each named emotion, independent coders interpreted the reasons for its appearance, grouping these explanations by content. In addition, the effect of the patient's named disease-related events on emotion was examined in terms of whether they strengthened, maintained, or weakened the intensity of emotions.

In analysing the post-test responses, we explored which subjective, psychosocial factors, other than the events of the disease process, played a role in shaping the emotion. Participant responses were also categorised into groups according to their content. Discussions were held among the researchers on the categories of events and categories of the content of events created and ensured that the data were free of personal biases. The italicized verbatim quotations were also used to underpin the findings of the study. Descriptive statistics were used to illustrate sociodemographic and clinical variables, which were performed using SPSS version 25.0 [37].

## Results

### Reasons for the appearance of emotions accompanying the disease process

We summarised the reasons patients gave for the emotions which accompanied the disease process, separating negative and positive emotions, which reasons were then identified by the participants.

### Reasons for the appearance of negative emotions

The reasons given by patients are presented by category for clarity, in each case where respondents attributed multiple causes to the emergence of the emotion. The most frequently portrayed reasons for feeling *fear* can be divided into three categories (persons with identification numbers 7, 8, 15, 19, 27, 32, 35, 36, 38, 40): (1) in most cases, the reason of fear is the unexpected diagnosis of breast cancer and thus its uncontrollability, (2) the damaging side effects of oncological treatments, and, in line with those patients (with identification numbers 3, 13, 14, 25) who presented *fear of death*, (3) the interpretation of malignant breast tumor as a fatal disease was used to explain the appearance of this emotion.

"I was afraid of the side effects and that the tumor would be inoperable. And at the end of treatment, I was afraid that the chemotherapy hadn't worked, that I wouldn't be able to be operated on." (Subject 19)

"I was shocked to hear the news, my parents died of the same disease (cancer)." (Subject 3)

"For me, cancer meant death." (Subject 25)

The presumed reasons for the emergence of *uncertainty* in the eight participants (with identification numbers 1, 10, 20, 21, 26, 31, 38, 42) were (1) doubt about the successful curability of the disease (2) the nature of cancer as a near-death experience, (3) delay in the treatment process due to ongoing Covid-19 pandemic, (4) uncertainty of patient suitability for surgery, and (5) the difficulty of communicating with the social environment.

"I felt insecure about whether the physicians could cure me." (Subject 20)

„Due to circumstances that hindered my recovery, the delays and waiting made me feel increasingly uncertain.” (Subject 1)

„I couldn't bear the uncertainty until I had reliable information; I felt insecure.” (Subject 42)

Patients who presented feelings of *anxiety* in their graphs (persons with identification numbers 4, 37, 38, 39, 40) attributed the dominance of this emotion to reasons that could be classified into two categories: (1) the unexpected arrival of the disease and unpredictability of the future, and (2) the underlying trait-level anxiety in the patient's personality.

"I couldn't help myself, I resisted the situation. I didn't know how to help myself." (Subject 4)

"I'm very anxious anyway, I get stressed about everything." (Subject 37)

In the graph of the emotional changes during the disease process, three patients (persons with 2nd, 6th, and 7th identification numbers) showed *anger* as the most dominant emotion accompanying the disease process, which has been explained by two patients as a reaction to the helplessness they felt when they were diagnosed with a malignant breast tumor (persons with 2nd & 7th identification numbers), and another patient felt anger about the waiting and uncertainty of admission to the clinic (person with 6th identification number).

The two patients (14 & 41 identification numbers) who reported a feeling of *helplessness* as the most dominant emotion during their disease process explained the occurrence of this feeling in one case as a feeling of withdrawal associated with the fear of death (Subject 14), while the other patient explained the feeling by the prolonged and long periods of ineffective and unsuccessful examination processes (Subject 41).

*Panic* and *shock* were reported in case of two subjects, the reasons for which were asymptomatic but severe illness (Subject 6) and the unexpectedness of the illness (Subject 41). The negative emotions that appeared once in the study sample were tension (cause: being vulnerable), alienation (cause: unexpected diagnosis), disappointment (cause: loss), and surprise (cause: denial due to the sense of inviolability).

### Reasons for the appearance of positive emotions

The feeling of *hopefulness* appeared as a dominant, positive emotion in six disease processes (persons with 12, 17, 27, 32, 33, and 42 identification numbers), the reasons given by the subjects included (1) knowledge of the curability of breast cancer, (2) seeing all events related to the cure as a step forward, (3) and an optimistic attitude.

"I was optimistic because I knew that breast cancer could be cured! And I knew I would be in good hands."(Subject 12)

"There was always a next step, a signal that gave me a boost." (Subject 17)

As the dominant emotion accompanying the disease process, two participants reported the feeling of *trust*, which was explained by one patient as a feeling of self-efficacy, searching for and finding solutions to help healing (Subject 5). In contrast, the other patient (Subject 30) attributed it to the experience of trust for the healing professionals.

"Yes, I can do it for myself!" (Subject 5)

"I felt myself in very good hands." (Subject 30)

The feeling of *calmness* was also the most dominant emotion felt by two patients (9 & 24 identification numbers) during the pathway of their illness, the reason given by Subject 9 for feeling this way was that she did not feel ill, so she was able to remain calm, and Subject 24 was able to remain calm all the time because she felt that there was a solution to her problem.

„I was able to stay calm because I knew there was a solution to my problem.” (Subject 24)

The emotion of *determination* was also the most dominant in two patients (with identification numbers 22 and 29) during the course of their illness, due to confidence in medical intervention and the emergence of a desire to recover.

"All the time I felt that every examination was helping me and moving my issue forward!" (Subject 22)

The feeling of *pleasure* as the most dominant emotion during the course of the disease was observed in two patients (with 23 & 28 identification numbers), Subject 23 explained her feeling by the fact that she had always been looking for handholds during the course of her illness, which made her feel she was going through a psychological development, and the 28th Subject explained that the onset of the disease was preceded by a symptomatic, painful period, so the healing process marked the end of this difficult period.

"For me, it was 8 months of spiritual education." (Subject 23)

The positive emotions that emerged once in the study sample were gratitude (cause: positive thinking), willpower (cause: seeing recovery as a task), and acceptance (cause: trust in recovery).

### Emotion change dynamics based on the produced emotion graphs

Our main research question was: how do the events that the patient considers important affect the emotions that accompany the disease process? In other words, which of the events associated with the healing process increase, or decrease the intensity of negative or positive emotions in women with breast cancer? In the following sections, we discuss the events that enhance, or weaken the intensity of negative, or positive feelings.

### Events that intensify negative emotions

According to our analysis, the events that intensify and maintain negative feelings include (() indicates how many people mentioned this event): the first detection of the tumor (25), the process of investigation (22), waiting for test results and then being confronted with an accurate diagnosis (26), managing examinations and time (15), chemotherapy (10), waiting for surgery (16), the process of surgery and anesthesia and the pain experienced afterwards (23), and changes in body image (17).

"There was the fear of what if the result is wrong?" (Subject 36: intensity of fear feeling increases from 20 to 30%; event: examinations)

"Again the questions and uncertainty about what to do next?" (Subject 38: intensity of feeling of uncertainty increases from 20 to 50%; event: present—following day after surgery)

"I took a cold shower, I didn't come to my senses for a day" (Subject 3: intensity of fear of death 50%; event: diagnosis)

"The wait for the results was unbearable! The Onco Team first decided to include chemotherapy before surgery, which I did not agree to, I was very afraid of it" (Subject13: intensity of fear of death in both events 70%; events: PET CT and decision of the Onco Team)

"I cried continuously until the PET CT, I was living all the negative stress" (Subject 14: intensity of fear of death 100%; event: PET CT)

"I can so see that ultrasound image in front of me, my breasts are full of lumps! I scanned the internet helplessly, reading up on everything." (Subject 41: intensity of feeling of helplessness 100% for both events; events: breast ultrasound and time of examinations)

"The sight of what I looked like during chemotherapy was very upsetting for me and my family. The treatments made me feel very weak." (Subject 35: intensity of feeling of fear increases from 70 to 80%; event: chemotherapy)

"I was expecting something else…I freaked out when I looked in the mirror for the first time!" (Subject 39: intensity of anxiety increases from 80 to 100%; event: first look in the mirror after surgery)

### Events that diminish the intensity of negative emotions

Several events contributed to the relief of negative feelings: reassuring information about cancer and treatments that pointed towards a cure (27), the end of chemotherapy (10), and successful surgery as an important step towards the possibility of a cure (21), which meant relief and freedom from disease for the subjects.

"Before the operation, I felt calmer, I knew I was going to have the operation, and the problem would be solved." (Subject 19: fear intensity reduced from 100 to 50%; event: surgery—mastectomy)

"Today, after the operation, it is a great relief, I feel that I am back to my old self again, I have got rid of what does not belong in me." (Subject 36: intensity of fear decreased from 30 to 0%; event: post-operative state)

"By this time, the uncertainty had diminished, the tumor had shrunk, the operation was next." (Subject 20: intensity of feeling of uncertainty reduced from 100 to 70%; event: last chemo)

"The end of chemotherapy meant a huge release." (Subject 31: intensity of feeling of uncertainty reduced from 90 to 50%; event: end of chemotherapy)

"When I arrived for surgery I was already calm, I knew that this was the solution." (Subject 3: fear of death intensity reduced from 15 to 5%; event: surgery)

"It is a great relief for me to have the tumor removed from my body." (Subject 13: fear of death reduced from 50 to 20% intensity; event: surgery)

"My fears were greatly reduced when I was faced with the fact that there was no metastasis, no high tumor markers, so I could relax. And with surgery, it was complete peace of mind." (Subject 14: intensity of fear of death reduced from 100 to 0%; event: surgery)

### Events that reduce the intensity of positive emotions

Events that can reduce the intensity of positive emotions include: receiving an accurate diagnosis (10), attending a biopsy for fear of confirmation of an aggressive tumor (8), undergoing breast surgery because of increased feelings of vulnerability (6), receiving negative test results (12), postponing surgery (4), starting chemotherapy (7), and the whole process of chemotherapy (6).

"I hit rock bottom after the fourth chemo, that's when I felt the worst physically! Just going to Oncology made me feel bad." (Subject 23: pleasure intensity decreased from 100 to 0%; event: “rock bottom")

"As I approached surgery, I felt more and more helpless." (Subject 5: intensity of feeling of trust decreased from 80 to 60%; event: surgery)

### Events that amplify positive emotions

Among the events that reinforce the intensity of positive emotions, we can include scans as an event interpreted by patients as a step forward in the healing process (9), chemotherapy as an intervention interpreted by patients as an important part of the healing process (4), tumor shrinkage (7), completion of chemotherapy (8), surgical removal of the tumor as a medical intervention interpreted as an important milestone in the treatment (5), surgical consultations (6), turning to alternative medicine and spirituality (3).

"I could feel that it was progressing. The tumor has been removed, I feel relieved, I have no choice but to get better, and I know I will get better!"(Subject 12: intensity of feeling of hope increases from 50 to 70%; event: surgery)

"My aim with meditation was to experience the present better, not to always want to control everything. Then I started looking into alternative medicine. I started taking supplements, plus I got a lot of encouragement and motivation." (Subject 5: intensity of feeling of confidence increases from 70 to 75%, then to 85%; events: meditation and practicing alternative medicine)

"When the PET CT result arrived and it was confirmed that everything was negative, no metastases, I cried for two hours with delight!" (Subject 29: intensity of feeling of determination increased from 30 to 90%; event: PET CT result: all negative)

"For me, surgery is closure, the end of the 8 months. I now feel that I don't have cancer." (Subject 23: pleasure intensity 100%; event: breast surgery)

### Illustrating the change through the example of a positive emotion

**Fig. 1:**
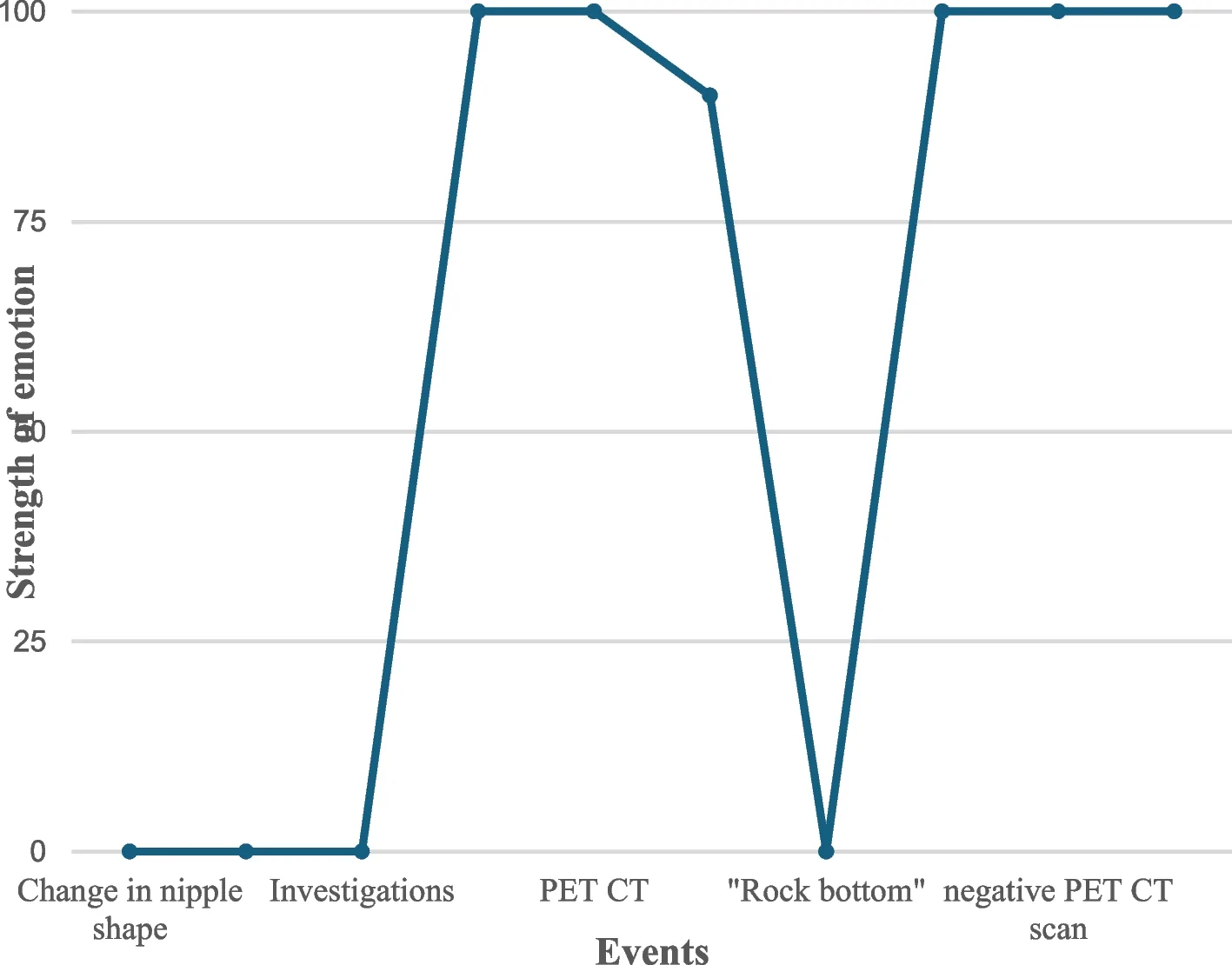
Digitized version of the Emotional Graph of Illness Trajectory prepared by Subject 23. The graph shows the change in intensity of the feeling of pleasure accompanying the disease process

The change in the feeling of pleasure is plotted on the Emotional Graph of Illness Trajectory prepared by Subject 23 in Fig. 1.

A prominent feature of the graph is that the intensity of pleasure ranges between extremes: either the feeling disappears completely or it is very intense. The patient associated an intensity of pleasure of 0% to the initial steps of the investigation, which were used to establish the exact diagnosis, and also to a "low point" experienced during chemotherapy. However, during events that led to recovery, such as an examination, therapeutic intervention, breast operation or religious ritual, the patient reported a feeling of pleasure of 90 and 100% intensity.

### Other subjective factors affecting changes in emotions

As a closing part of the Emotional Graph of Illness Trajectory taken with the subjects, the post-test questions ("*What did the patient do to change the low/high points?* ") provide insights into how the patient contributed to the reduction of negative feelings and the increase of positive feelings, i.e. what adaptive methods she used to regulate her emotional state, and how she contributed to the increase of negative feelings and the reduction of positive feelings, i.e. what maladaptive methods she used to regulate her emotions.

The post-test question on what the patient actively did to alleviate negative emotions (i.e. to reduce the intensity of the strong emotional intensity scores on the graph) resulted in the following response categories (we indicate in parentheses what percentage of the sample mentioned this factor): (1) trust in the treatment plan (60%), (2) contact with breast cancer survivors (30%), (3) development of self-awareness, consultation with a psychologist (25%), (4) lifestyle changes (diet, physical activity) (35%) and (5) turning to spirituality (20%). When asked about the patient's contribution to the intensification of negative emotions (i.e., the increase in intensity of the points on the graph indicating weak emotional intensity), the following response categories were obtained: (1) the presence of anxiety as a personality trait (30%), (2) time as a stressor: worry that she would run out of time (35%), (3) lack of confidence in recovery (40%).

"I meditated a lot, I prayed, I read. I tried to heal myself." (Subject 10)

"I've thought things through carefully. I had to learn to accept the support of security, to change my values and expectations." (Subject 20)

According to post-test results, the following response categories were obtained when asked about what the patient did to increase the positive emotion (i.e., to increase the intensity of the points on the graph indicating weak emotional intensity): (1) belief in and trust in doctors and treatment (40%), (2) experiencing the success of the cure (55%), (3) lifestyle changes (25%), (4) turning to spirituality (25%), (5) interpreting the disease as a solvable problem (25%), (6) excluding internet resources (10%), (7) practicing stress reduction techniques (15%). In response to the question of how the patient contributed to the weakening of positive emotion (i.e. the reduction in the intensity of points on the graph indicating strong emotional intensity), according to the responses received, patients felt a weakening of positive emotions due to the way they reacted to disease-related negative changes (over-involvement), and not seeking a solution to cope with the situation.

### Patients' experiences of the Emotional Graph of Illness Trajectory test situation

In the final phase of the post-test, we asked participants what it was like to create an emotional graph of their disease process. Patient responses were summarised as follows: (a) they were able to think about their disease process from a new perspective, not the usual one; (b) it was a new viewpoint to think about the emotional impact of events and to observe the changes in their emotional state in the form of a graph; (c) and last but not least, the previously fragmented story of the disease could be transformed into a coherent perspective about the illness through the graph.

## Discussion

In our exploratory descriptive qualitative research, we aimed to explore the event-related emotional experiences during the illness trajectory of women with breast cancer in the postoperative state. The Emotional Graph of Illness Trajectory was used to identify the dominant emotions accompanying the disease process in 42 women diagnosed with breast cancer and to relate changes in these emotions to events during the healing process. According to our results, only a small proportion of the study sample (26.19%) had positive emotions associated with their disease process, while the majority had negative emotions on their graph. According to medical records, patients reporting positive emotions did not differ from patients reporting negative emotions in terms of elapsed time from diagnosis, chemotherapy, and various breast surgery procedures. Our findings on the charge distribution of emotion expression are consistent with previous qualitative research findings that emotional reactions of breast cancer patients are overwhelmingly characterised by uncertainty, despair, hopelessness, and fear of death [1, 38, 39].

Further interpretation of the results outlined that the causes of negative and positive emotions reveal pairs of opposites (Table 2). These differ in their interpretations of diagnosis and medical treatment, and in terms how the patient views herself and her internal resources. Exploring the causes and the pairs of opposites highlights the importance of the emotion regulation process.

**Table 2:** Summary of causes behind the emergence of negative and positive emotions

Causes of the appearance of negative emotions	Causes of the appearance of positive emotions
- unexpected, onerous diagnosis - interpreted as incurable, fatal disease	- the disease is curable - denial of patient role
- burdensome oncological treatments - treatment-related inertia, uncertainty, waiting	- medical treatment is an important step towards recovery - trust in healing professionals and healing
- trait-level anxiety	- optimistic attitude - self-efficiency - the will to heal

However, it is a well-established fact in clinical work and literature that some patients are observed to view their cancer as a positive, challenging event that helps them maintain a positive self-image and manage negative emotions related to the disease and treatment [16, 22, 38, 40–44]. In our sample, positive emotions may have resulted from successful emotion regulation and a sense of post-traumatic growth, as patients who associated positive emotions with the disease process demonstrated a sense of self-efficacy and interpreted interventions related to the disease as a sense of achievement or completing a task. Another reason for the emergence of positive emotions was the denial of patient role, where the patient ignored the distress caused by the illness. Nevertheless, literature suggests that this technique is only effective on short-term while the patient gathers resources, but it impedes long-term coping [45–48].

**Fig. 2:**
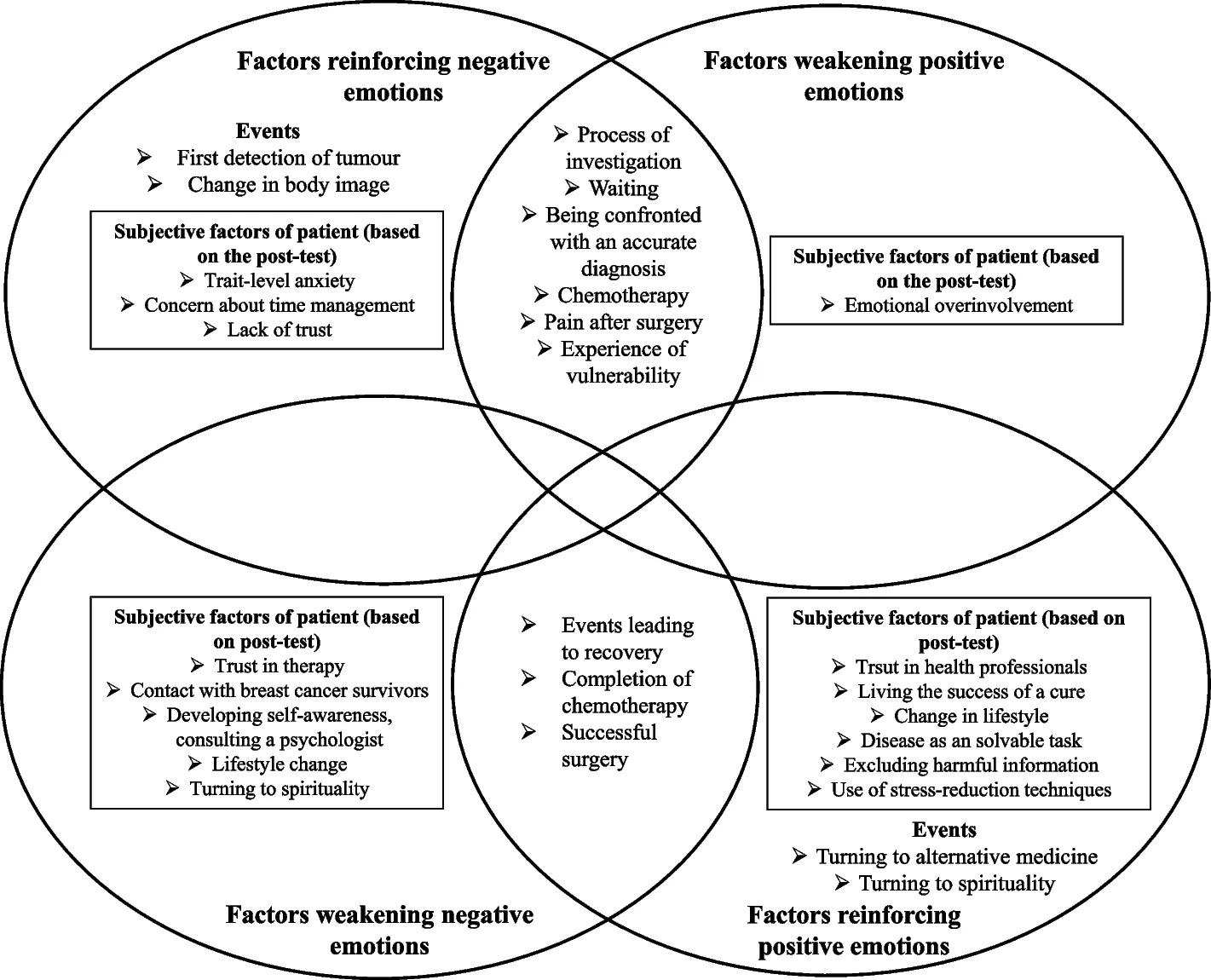
Summary of factors that increase and decrease the intensity of negative and positive emotions

The main highlight of the study is that the intersections of factors that influence the intensity of negative and positive emotions during the disease process include many of the same events. For example, an event increased negative affect in one person but decreased positive affect in another. This illustrates the importance of emotion regulation process (Fig. 2). As shown in the figure, certain events—such as being investigated for breast cancer, waiting for appointments and results, facing an accurate diagnosis, undergoing chemotherapy, experiencing post-operative pain, and feeling vulnerable—were responsible for both increasing negative- and decreasing positive emotions. Only negative emotions were reinforced by the initial tumor detection and the change in body image. Consistent with previous research, women often worry about body changes due to breast surgery; body image issues are present from the first signs and at the time of initial contact with the medical system through post-treatment [8, 49–51]. Intersections were also identified between events that reinforced positive emotions and those that diminished negative ones, including medical consultations, interventions (such as chemotherapy and surgery), and recovery outcomes (Fig. 2). It was only in relation to positive emotions that events like spirituality and turning to alternative medicine were described as factors that enhanced positive feelings. In the figure, the rectangles depict the subjective factors respondents mentioned during the post-test concerning changes in emotional state (e.g.: lifestyle adjustments).

Events that induce negative changes in emotion regulation (i.e. events that intensify negative emotions and weaken positive emotions) are typically associated with the initial phases of the disease, which have a distress-increasing effect, and consequently, negative emotions dominate the patient’s emotional process related to the disease during this period [22]. In contrast, events that promote positive emotion regulation (i.e. events that weaken negative emotions and strengthen positive emotions), can be linked to more advanced disease process, during which the patient can formulate a positive vision of the future [22, 40].

The analysis of patient responses showed that the development and change of feelings related to the disease were influenced both by how the patient perceived the illness and healing process and by which meaning is assigned to the event. We observed that, in one case, surgery signifies vulnerability and pain, while for another, it represents the possibility of removing a malignant tumor, making surgery a crucial step toward recovery. Cognitive change is an important emotion regulation strategy for cancer patients, involving the modification of appraisals of the situation, which is known as cognitive reappraisal, a part of the affect generation process [22, 52]. This strategy has generally been effective in reducing negative emotions [53–55]. Additional research shows it is especially useful when faced with unavoidable, targeted stimuli or situations [56]. This approach is particularly significant for cancer patients during the initial diagnosis and treatment phases, as they confront a potentially life-threatening condition. Using adaptive cognitive reappraisal strategies (as opposed to anxious worrying and rumination) has been linked to less psychological distress during the first year after cancer diagnosis and treatment across various cancer groups [57, 58]. Studies by Troy and colleagues [48] also confirm that reframing helps individuals cope with uncontrollable stress, such as a diagnosis of a malignant tumor, by reducing depressive symptoms. Reframing can be achieved through techniques like (1) changing the meaning of the situation (reinterpretation) or (2) creating psychological distance from the emotional situation (distancing) [59]. In our study, a good example of distancing is the patient's denial of their role, whereas reinterpretation involves believing that treatments and surgeries can bring the patient closer to full recovery.

Results also show that encouraging investigations, medical reports and positive illness-perception of the patient (e.g. a positive mental representation of surgery, helping the patient towards recovery) have contributed to the weakening of negative emotions and the strengthening of positive emotions in several patients. Our findings in connection with the mental representation of illness—for example, interpreting illness as incurable, resulting in negative emotions—were also consistent with previous research findings, suggesting that adverse changes in illness perceptions among patients with chronic illness were associated with increased negative emotions, negative changes in perceived consequences and perceived control over the illness [60, 61]. The results of an earlier study with amyotrophic lateral sclerosis also showed a correlation between patient disease- and mood representations, and that psychological factors also significantly determine the adaptive or maladaptive nature of patient disease-adaptation [62]. Our findings align with existing literature on the psychological challenges faced by breast cancer patients, e.g.: other qualitative analysis also reveals that body image concerns begin with initial diagnosis and are deeply intertwined with emotional responses, including fear, uncertainty, and ongoing struggle to maintain a sense of femininity [8, 63, 64].

## Conclusions

Our results emphasize the importance of early psychological interventions because positive reframing tends to be more effective in the initial stages than later when confronting a breast cancer diagnosis [55, 65–67]. Properly regulated emotional processes play a crucial role in successfully adapting to environmental challenges. From a therapeutic perspective, it is particularly noteworthy that, regardless of the method used, changes in emotion regulation during psychological interventions are often associated with changes in symptoms [68–70]. Given the dynamic nature of cancer, the flexibility of emotion regulation may be most effective for coping with the disease, rather than relying on a single regulation strategy. Some techniques, such as denial and distancing, may be helpful early on, but could be less effective later in the healing process [21, 22, 71–73].

According to a summary study by Stanton and Low [74], in expressing emotions, both verbally and in writing, we label and attribute our emotions. This psychological process can reduce the subjective intensity of the emotion experienced and help us understand and reframe the triggering stressor [74]. Our results align with previous findings, highlighting the importance of exploring the emotional aspects accompanying the disease process, as the emotional perspective provides insights into coping styles during this process [75]. In summary, storytelling can help restore a sense of personal control and reduce feelings of suffering, which are intrapersonal needs that are fundamentally damaged when facing a severe illness [76, 77].

Furthermore, data collected by the Emotional Graph of Illness Trajectory has implications for the development of modern psychological interventions. The detailed mapping of emotional changes could provide crucial input for Artificial Intelligence applications in healthcare [78]. In addition, interventions such as our graphic technique, which focus on the body, illness and emotional experience, align well with emerging tools such as Virtual Reality, which offers a powerful tool for promoting interoceptive awareness and improving body image among breast cancer survivors [15].

### Benefits of using of the Emotional Graph of Illness Trajectory

The presented graphic technique can help put the experience of illness into perspective, restructure, and re-evaluate through remembering, and thus restore the disconnectedness of life history, which can lead to a psychological process that adaptively contributes to reducing the traumatic impact of illness [32, 79–81]. The main advantage of the Emotional Graph of Illness Trajectory developed by our team is that it helps reconstruct the relationship between time, events, and emotions associated with illness, enabling a coherent description of illness. The tool has a communication-facilitating role that can enhance the effectiveness of psychological intervention in somatic patient care, helping patients verbalize distressing emotions caused by illness and fundamentally helping them develop their ability to communicate about their illness. The technique was well received by patients and can increase the patient's sense of control over the situation, thereby reducing anxiety in the test-taking situation and increasing the richness of response.

### Limitations

Limitations of our study include, firstly, that the sample was not homogeneous in terms of the therapeutic process; in addition patients differ in terms of elapsed time from diagnosis and content of therapeutic process (neoadjuvant oncological treatment, type of breast surgery), which may result in differences in the emotional processing of patients, furthermore it may also contribute to the development of recall bias, since recollection of events which occurred some time ago may be less accurate among individuals whose treatment began more than one year ago. Secondly, the focus on the events of the disease process may have limited the presentation of other perspectives on disease experience.

## Supplementary Information

Additional file 1: Supplementary material_Lévai et al. The additional file contains the instruction and the grid graphic of the Emotional Graph of Illness Trajectory and the marking sheet.

## Data Availability

The datasets generated and analysed during the current study are available in the Open Science Framework repository, [URL: https://osf.io/b8rux/].
