# Supplementary material for: On the pathway of changing emotions—a qualitative study of emotional changes in breast cancer patients from the first detection of the tumor to breast surgery
Source: BMC Womens Health. 2026 Aug 14;26:598. doi: 10.1186/s12905-026-04776-6 (PMC13749676; doi:10.1186/s12905-026-04776-6)
Supplement: Supplementary file 1 [file 12905_2026_4776_MOESM1_ESM.docx]

**On the pathway of changing emotions - a qualitative study of emotional changes in breast cancer patients from the first detection of the tumor to breast surgery**

Supplementary Material

Tünde Lévai^*^, György Lázár, Attila Paszt, Zsolt Simonka, Zoltán Horváth, Aurél Otllakán, Melinda Látos

*** Correspondence:** Corresponding Author: [tundelevai96@gmail.com](mailto:tundelevai96@gmail.com)

# 1 Supplementary information about the visual elicitation technique

## 1.1 Emotional Graph of Illness Trajectory:

- The instruction and the grid graphics
- The marking sheet

Please, draw a graph showing the emotional changes in your disease process on the grid below! You can mark the events along the horizontal axis and the strength of the emotion along the vertical axis. Choose one dominant emotion you have experienced during the course of your illness! Please mark the events that stand out for their emotional impact on the horizontal axis (e.g. diagnosis, treatments, medical tests, surgery), at a point of your choice, in chronological order, then rate and mark along the vertical axis the intensity of the emotion experienced at the time of the event! If you can link several crucial emotions to the disease process, their illustration on graphs (according to the criteria indicated above) is feasible in the same grid area!

***Strength of emotion***

0%

50%

100%

***Events***

**Emotional Graph of Illness Trajectory**

*Marking sheet*

| *Patient’s identifier:* | | *Diagnosis:* |
| --- | --- | --- |
| *Gender:* | *Birth date:* | *Disease-related intervention:* |

| **Emotion:** | | |
| --- | --- | --- |
| **Justification:** | | |
| 1. **Event** | | |
| **Denomination** |  | |
| **Describe of event** |  | **% value of emotional intensity:** |
| 2. **Event** | | |
| **Denomination** |  | |
| **Describe of event** |  | **% value of emotional intensity:** |
| 3. **Event** | | |
| **Denomination** |  | |
| **Describe of event** |  | **% value of emotional intensity:** |
| 4. **Event** | | |
| **Denomination** |  | |
| **Describe of event** |  | **% value of emotional intensity:** |
| 5. **Event** | | |
| **Denomination** |  | |
| **Describe of event** |  | **% value of emotional intensity:** |
| 6. **Event** | | |
| **Denomination** |  | |
| **Describe of event** |  | **% value of emotional intensity:** |
| 7. **Event** | | |
| **Denomination** |  | |
| **Describe of event** |  | **% value of emotional intensity:** |
| 8. **Event** | | |
| **Denomination** |  | |
| **Describe of event** |  | **% value of emotional intensity:** |
| 9. **Event** | | |
| **Denomination** |  | |
| **Describe of event** |  | **% value of emotional intensity:** |
| 10. **Event** | | |
| **Denomination** |  | |
| **Describe of event** |  | **% value of emotional intensity:** |
| Sum of percentages of emotional intensity associated with events (∑%): ____________  Number of events (db): ____________  Quotent of values (∑%/db): ____________ | | **Mean value of emotion alintensity:** |

**Post-test**

1. *What could have caused the graph to rise?*
2. *What could have caused the graph to fall?*
3. *If there were turning points, what caused them?*
4. What did the patient do to change the low points?
5. What did the patient do to change the high points*?*
